# Development and validation of the acculturation discrepancy scale for youth of Korean descent in Japan (ADS-KJ)

**DOI:** 10.1186/s40359-026-03998-5

**Published:** 2026-04-09

**Authors:** Misa Sasaki, Chiharu Maeda, Tomu Ohtsuki, Taisuke Katsuragawa

**Affiliations:** 1 Department of Clinical Psychology, Graduate School of Human Sciences, Waseda University, 2-79-15 Mikajima, Tokorozawa, Saitama , 359-1192 Japan; 2 Faculty of Human Sciences, Waseda University, Tokyo, Japan

**Keywords:** Korean descent in japan, Acculturation discrepancy, Scale development

## Abstract

**Background:**

This study aimed to develop and validate the *Acculturation Discrepancy Scale for Youth of Korean Descent in Japan* (ADS-KJ). The scale measures youths’ subjective perceptions of intergenerational cultural gaps, that lead to parent–child acculturation discrepancies.

**Methods:**

Based on interview data and a review of previous studies, an item pool was created and administered via questionnaire surveys to 278 youths of Korean descent in Japan and 110 Japanese youths. The scale’s structural validity, construct validity, internal consistency, test-retest reliability, and criterion-related validity were examined.

**Results:**

Consequently, it was developed as a 21-item, two-factor scale comprising *Identity Discrepancy* and *Values Discrepancy*. ADS-KJ demonstrated generally good reliability and validity, though some areas relating to its criterion-related validity need further examination.

**Conclusion:**

As a culturally sensitive assessment tool, ADS-KJ shows potential to capture youths’ subjective perceptions of parent–child acculturation discrepancies in families of Korean descent in Japan. Thus, it can guide and support Korean descent youth in Japan’s growing immigrant society.

**Supplementary Information:**

The online version contains supplementary material available at 10.1186/s40359-026-03998-5.

## Background

 Acculturation is a developmental process in which individuals come in contact with and adapt to a new culture [1], most commonly in the context of immigration [2]. In particular, in immigrant families, parents and children may experience intergenerational acculturation differences, also known as acculturation gaps, whereby they each hold discrepant cultural views or degrees of cultural adaptation as it relates to the receiving culture and/or their heritage culture [3]. The acculturation gap-distress model purports that immigrant children acculturate faster to the new host culture than their parents do, and family conflict and youth maladjustment arise as a result [4]. Several recent studies have provided support for this model [5–8]. For example, Kim and Park [5], in a sample of 77 Korean American parent–child dyads, demonstrated that greater acculturation gaps were associated with higher levels of depressive symptoms, anxiety, and aggression among youth. Similarly, Rahman et al. [6], in a study of 200 Hispanic Americans, found that acculturation gaps were significantly associated with poorer self-rated health.

However, two main issues remain in previous studies on acculturation gaps. First, limitations exist in their measurement methods. Traditionally, the acculturation levels of parents and children have been measured separately, and the numerical differences between them have been used as an indicator of acculturation discrepancy [9]. This approach assumes that the gap between acculturation levels objectively reflects an acculturation discrepancy. However, even when such a gap exists, if youth are not consciously aware of this discrepancy, its psychological impact may be minimal. Rather, it is their subjective perception of the acculturation discrepancy that may influence their psychological adjustment more strongly [6, 10, 11]. For example, Smokowski et al. [10] found that acculturation gaps calculated from parent–child reports were not associated with family functioning, whereas youths’ perceived acculturation discrepancies were significantly related to poorer family functioning. Despite this, no standardized measure has been developed to assess youths’ subjective perceptions of acculturation discrepancies.

Second, a limitation exists regarding previous studies’ target populations. The effects of acculturation discrepancy on youths tend to be shaped by each immigrant group’s unique migration [5]; therefore, detailed studies for each cultural group are needed. However, most prior research has focused on Hispanic and Asian American immigrant populations in the United States [8], and little is known about acculturation discrepancies in other cultural contexts. In the Japanese society, where the immigrant population has been growing rapidly in recent years, research on this topic remains particularly limited.

Among the diverse immigrant populations in Japan, youth of Korean descent exhibit the highest suicide rates [12]. This phenomenon is rooted in discriminatory structures and parent-child conflicts influenced by the legacy of colonial rule [13]. The Korean Peninsula was under Japanese colonial rule from 1910 to 1945. Japan adopted “assimilation” as a basic policy during this period. While this policy ostensibly aimed to make Koreans indistinguishable from the Japanese people, it did not imply treating them as equals with the same rights [14]. Immediately following colonization, Koreans were unilaterally granted Japanese nationality and forced to worship at shrines, raise the national flag, use the Japanese language, wear traditional Japanese clothing, and adopt Japanese names [15, 16]. School enrollment rates for Korean children were low, and those who did attend faced discrimination from classmates, such as being told they “smelled like kimchi” or to “go back to Korea” [16]. With Japan’s defeat on August 15, 1945, colonial rule ended. Simultaneously, approximately two million liberated Koreans established autonomous organizations across the country, which later became ethnic associations [15]. Even today, 80 years later, in a Japanese society where imperial consciousness persists, individuals of Korean descent continue to be subjected to discrimination and hate speech [17].

Against this historical backdrop, youths of Korean descent in Japan are generally categorized into two subgroups: Oldcomers and Newcomers. Oldcomers refer to individuals, who either directly or through their family lineage, were affected by Japan’s colonial rule of Korea between 1910 and 1945, had migrated to Japan either during that period or shortly after, and whose descendants now span multiple generations. This group includes individuals with Korean, Joseon, Japanese, or multiple national affiliations, including those born into multiethnic families through intermarriage [18]. In contrast, newcomers refer to those who had migrated to Japan since the 1980s for reasons, such as employment, international marriage, education, or along with their children [19].

Among oldcomers, a commonly observed form of acculturation discrepancy involves differing perceptions of affinity toward Japanese society. Parents in this group, shaped by their experiences with the lingering structures of colonial era discrimination, often hold a cautious or distrustful view of Japanese society [20]. In contrast, many of their children, who have been born and raised in Japan, and often have little to no experience with Korea [21, 22], tend to develop a greater sense of affinity and positive orientation toward Japanese society through their schooling and peer relationships. This divergence between parental distrust and children’s affinity toward Japanese society may manifest as an acculturation discrepancy. Such dynamics can lead to increased parent–child conflict, which in turn, has been linked to adverse outcomes for youths, such as ethnic identity diffusion and interpersonal hypersensitivity [23].

Among newcomers, a commonly observed form of acculturation discrepancy involves differences in Japanese-language proficiency between parents and children. Many second-generation or later newcomer youths are born in Japan and speak Japanese as their first language. In contrast, their parents who have immigrated more recently often have limited proficiency in Japanese. This discrepancy in language skills can lead to role reversals within the family, as children may take on responsibilities, such as reading school communication and translating it for their parents [21]. Such dynamics may contribute to role conflicts for the children, and have been found to increase their risk of psychological difficulties, including depression and anxiety [24]. However, to date, no standardized measure exists to assess subjective perceptions of acculturation discrepancies in this population, and empirical studies examining these associations are still lacking.

This study aimed to develop and validate a scale to assess youths’ subjective perceptions of acculturation discrepancies in Korean descent families in Japan. In addition, it examined discriminant validity by including a comparison group of Japanese youth to confirm that the scale captures acculturation discrepancies specific to immigrant family contexts. It is expected to convey the development of culturally informed psychological support for youth of Korean descent in Japan by providing a tool to assess subjective perceptions of acculturation discrepancies.

## Methods

### Item pool development

The initial item pool for this study was developed through qualitative interviews with 12 youths of Korean descent in Japan, each of whom took part in a semi-structured interview lasting an average of 1 h and 3 min (*SD* = 15 min 48 s) focusing on their perceptions of acculturation discrepancies. They were recruited through student and youth groups organized by ethnic organizations and through snowball sampling. The interview protocol included six open-ended questions ( e.g., *“In what situations have you recognized an acculturation discrepancy?”* and *“How did you feel in those situations?”*).

Data from the interviews were analyzed using the Jiro-Kawakita method [25], a qualitative analysis technique widely used in Japan to organize conceptual structures from grouped data [26], and frequently employed in item development for psychological scales [27, 28]. Participants received a stipend of 1,500 yen for their participation.

Based on the interview findings and a review of existing acculturation measures— such as the Vancouver Index of Acculturation (VIA) — [29], an initial pool of 45 items was created in collaboration with one faculty member specializing in clinical psychology and two graduate students in the same field. Subsequent revisions were made based on cognitive interviews with five individuals of Korean descent in Japan, which led to modifications in item wording and the removal of 20 items. An additional round of item refinement was conducted through an e-Delphi survey [30] involving 15 individuals of Korean descent in Japan, in which participants rated the importance of each item on a 5-point scale (from *“not important at all”* to *“very important”*), and provided open-ended feedback regarding item content. This process resulted in further revisions to item wordings and the removal of two items, yielding a final item pool of 23 items. The resulting items covered two key domains: *Identity Discrepancy*, which assessed perceived differences in cultural affiliation (e.g., *“I perceive differences with my parents regarding which cultural background I would prefer my marriage partner to have”*) and *Values Discrepancy*, which captured differences in cultural values, particularly those related to Confucian traditions (e.g., *“I perceive differences with my parents regarding the belief that men must succeed in society”*). These items were designed to reflect both multiple cultural affiliations beyond a single national identity and tensions related to traditional Confucian values.

### Participants and data collection

From July 2023 to March 2025, questionnaire surveys were administered to youths of Korean descent in Japan and Japanese in Japan. The surveys were conducted either online or in person. In line with prior definitions of emerging adulthood that extend the adolescent period up to approximately age 30 , participants were aged 18–30 years. Participants were recruited through three student and youth groups organized by ethnic community organizations, as well as through universities in the Tokyo metropolitan area. In the community organizations, recruitment was conducted during cultural exchange events by distributing flyers with QR codes linked to an online survey (Google Forms). The decision to recruit through ethnic community organizations was grounded in the finding that approximately 40% of households of Korean descent in Japan are affiliated with such organizations, which include individuals of diverse backgrounds such as “doubles” (multiethnic individuals), naturalized citizens, and newcomers [31]. We anticipated that this approach would enable access to participants with diverse migration histories. At universities, questionnaires were distributed to students before or after psychology lectures.

A total of 278 youths of Korean descent in Japan (*M* = 21.63, *SD* = 3.15, 133 women, 143 men, 3 chose not to answer) provided valid responses, and were included in the analyses of structural validity, internal consistency, test–retest reliability, construct validity, and criterion-related validity. In addition, data from 110 Japanese youths (*M* = 22.08, *SD* = 3.45, 63 women, 45 men, 1 chose not to answer) were used to examine discriminant validity. For the test–retest reliability analysis, a second survey was administered approximately four weeks after the first survey. Participants who had consented to follow-up were contacted via email using addresses collected from the initial survey’s demographic form. Participants’ identities were verified using the last four digits of their mobile phone numbers. Data from 45 youths of Korean descent in Japan who completed both surveys were used for test–retest reliability analyses.

## Measures

### Demographic variables

Participants reported their age, gender identity (male, female, or prefer not to answer), generational status, ethnic organization membership, and highest level of education completed. Generational status was classified according to the criteria outlined by Rumbaut (2002): fifth generation (from great-great-grandparents), fourth generation (from great-grandparents), third generation (from grandparents), second generation (from parents), 1.5 generation (born in Korea and immigrated to Japan before age 18), and first generation (born in Korea and immigrated after age 18) [32]. Participants selected their generations from these six categories, with an additional option of “unknown.”

### ADS-KJ

The ADS-KJ developed in this study consists of two subscales: *Identity Discrepancy* (11 items) and *Values Discrepancy* (12 items), which assess youths’ subjective perceptions of acculturation discrepancies between themselves and their parents. Each item is rated on a 5-point Likert scale ranging from 1 (*strongly disagree*) to 5 (*strongly agree*), with higher scores indicating greater perceived acculturation discrepancy.

### PHQ-9

The Japanese version of Patient Health Questionnaire-9 (PHQ-9) [33] was used to assess the severity of depressive symptoms. It consists of nine items corresponding to the diagnostic criteria for major depressive disorders. Participants rated how frequently they had experienced each symptom during the past two weeks on a 4-point scale ranging from 0 (*not at all*) to 3 (*nearly every day*), with higher total scores indicating a greater severity of depressive symptoms.

### GAD-7

The Japanese version of the Generalized Anxiety Disorder-7 (GAD-7) [34] was used to assess the severity of generalized anxiety symptoms. It consists of seven items that correspond to the diagnostic criteria for generalized anxiety disorders. Participants rated how frequently they had experienced each symptom during the past two weeks on a 4-point scale ranging from 0 (*not at all*) to 3 (*nearly every day*), with higher total scores indicating a greater severity of anxiety symptoms.

### UCLA loneliness

The Japanese version of the UCLA Loneliness Scale (Version 3) (UCLA Loneliness) [35] was used to assess the participants’ subjective perceptions of social isolation. The scale consists of 20 items rated on a 4-point scale ranging from 1 (*always*) to 4 (*never*). Following standard scoring procedures, the positively worded items were reverse-coded and summed so that higher total scores indicate higher social isolation. Its psychometric properties have been validated not only for older adults, but also for use with youth populations [36].

### VIA

The Vancouver Index of Acculturation (VIA) [29] was used to assess the participants’ acculturation levels. It consists of 20 items and comprises two subscales: *VIA–Heritage*, which measures adaptation to heritage culture, and *VIA–Mainstream*, which measures adaptation to mainstream culture. In this study, “heritage culture” was operationalized as Korean culture, and “mainstream culture” as Japanese culture. The Japanese version of the VIA was developed using back-translation procedures by two faculty members experienced in scale translations. Each subscale demonstrated good internal consistency (VIA–Heritage, *ω* = 0.88; VIA–Mainstream, *ω* = 0.80). Participants rated each item on a 9-point scale ranging from 1 (*strongly disagree*) to 9 (*strongly agree*), with higher scores indicating a greater adaptation to heritage or mainstream cultures, respectively.

The PHQ-9, GAD-7, and UCLA Loneliness Scale were used to examine ADS-KJ’s construct validity. These measures were selected based on findings from the qualitative interviews and prior studies indicating associations between acculturation discrepancies and internalizing symptoms in youth [8, 37, 38]. Additionally, VIA was used to examine ADS-KJ’s criterion-related validity, as previous research has suggested that acculturation discrepancies may arise when children exhibit high adaptation to mainstream culture and low adaptation to heritage culture [39]. VIA’s bi-dimensional structure permits the assessment of both heritage and mainstream cultural adaptations.

### Data analysis

To examine ADS-KJ’s structural validity, item–total correlations were first calculated to examine the consistency of each item with the total scale. The number of factors was estimated using the minimum average partial (MAP) correlation method. Exploratory factor analysis (EFA) was conducted using the minimum residual method with oblimin rotation, followed by confirmatory factor analysis (CFA) to evaluate model fit, which was assessed using the following indices: Comparative Fit Index (CFI), Tucker–Lewis Index (TLI), Standardized Root Mean Square Residual (SRMR), and Root Mean Square Error of Approximation (RMSEA). The following cutoff criteria were used to indicate an excellent fit: CFI and TLI ≥ 0.95, SRMR ≤ 0.08, and RMSEA ≤ 0.05 [40].

Internal consistency was evaluated by calculating McDonald’s *ω* coefficients for each subscale. Based on Dunn, Baguley, and Brunsden’s [41] study, values of *ω* ≥ 0.90 (excellent), 0.80–0.89 (good), and 0.70–0.79 (acceptable). Test–retest reliability was assessed using intraclass correlations (*ICC)* based on data from 45 participants of Korean descent in Japan, who completed both surveys. Interpretation of *ICC* values followed Koo and Li’s [42]. guidelines: ≥ 0.91 (excellent), 0.75–0.90 (good), 0.50–0.75 (moderate), and < 0.50 (poor).

Construct validity was examined by calculating the correlations between the scores on the two subscales of ADS-KJ and those on the PHQ-9, GAD-7, and UCLA Loneliness Scale among Participants of Korean descent in Japan. To examine discriminant validity, the same correlations were calculated among the Japanese participants. The interpretation of the correlation coefficients followed Guilford’s [43] guidelines: |*r*| > 0.70 (strong), 0.40–0.70 (moderate), and < 0.40 (weak).

Criterion-related validity was examined by calculating the correlations between the scores on the ADS-KJ and VIA subscales among participants of Korean descent in Japan.

### Hypotheses

Hypotheses were developed by referring to the consensus-based standards for the selection of health measurement instruments (COSMIN) guidelines for evaluating the quality of patient-reported outcome measures [44].

### Structural validity

The ADS-KJ would exhibit a two-factor structure consisting of *Identity Discrepancy* and *Values Discrepancy* (Hypothesis 1).

### Construct validity

Significant positive correlations would be observed between the scores on the two subscales of ADS-KJ and those on the PHQ-9, GAD-7, and UCLA Loneliness Scale among Participants of Korean descent in Japan (Hypothesis 2). No significant correlations would be observed among Japanese participants, supporting discriminant validity (Hypothesis 3).

### Criterion-Related validity

Among Participants of Korean descent in Japan, significant negative correlations would be observed between the ADS-KJ subscale scores and VIA–Heritage scores, and significant positive correlations would be observed between the ADS-KJ subscale scores and VIA–Mainstream scores (Hypothesis 4).

## Results

### Participants’ characteristics

Table 1 presents the participants’ demographic characteristics. Among Participants of Korean descent in Japan (*N* = 278), gender was approximately evenly distributed, and most had completed at least high school education. In terms of generational status, the fourth generation comprised the largest group (60.8%), followed by the third generation (21.9%). Additionally, 89.6% of Participants of Korean descent in Japan reported membership in an ethnic organization.

**Table 1:** Descriptive sample characteristics

Variable	Korean Descent sample (N ＝ 278)	Japanese sample (N = 110)
Age	21.63 (SD = 3.15) [18–30]	22.08 (SD = 3.45) [18–29]
Gender		
Women	133 (47.8%)	47 (42.7%)
Men	142 (51.1%)	62 (56.4%)
Prefer not to answer	3 (1.1%)	1 (0.9%)
Highest educational degree		
Junior high school degree	3 (1.1%)	0 (0%)
High school degree	204 (73.4%)	70 (63.6%)
Associate degree	11 (4.0%)	0 (0%)
Bachelor’s degree	49 (17.6%)	39 (35.5%)
Advanced degree	11 (4.0%)	1 (0.9%)
Generation status		
First generation	6 (2.2%)	–
1.5 generation	6 (2.2%)	–
Second generation	19 (6.8%)	–
Third generation	61 (21.9%)	–
Fourth generation	169 (60.8%)	–
Fifth generation	1 (0.4%)	–
Ethnic organization affiliation		
Affiliated	249 (89.6%)	–
Not affiliated	29 (10.4%)	–
ADS-KJ		
Identity Discrepancy	M = 1.82 (SD = 0.16) [1–5]	M = 1.30 (SD = 0.53) [1–5]
Values Discrepancy	M = 2.22 (SD = 0.16) [1–5]	M = 1.77 (SD = 0.76) [1–5]
PHQ-9	M = 0.82 (SD = 0.96) [0–3]	M = 0.83 (SD = 0.99) [0–3]
GAD-7	M = 0.78 (SD = 0.95) [0–3]	M = 0.81 (SD = 0.97) [0–3]
UCLA Loneliness	M = 1.95 (SD = 0.93) [1–4]	M = 2.08 (SD =0.56) [1–4]
VIA		
Heritage	M = 6.31 (SD = 1.63) [1–9]	–
Mainstream	M = 5.83 (SD = 1.28) [1–9]	–

Note. *ADS-KJ* Acculturation Discrepancy Scale for Youth of Korean Descent in Japan, *PHQ-9* The Japanese version of Patient Health Questionnaire-9, *GAD-7* The Japanese version of Generalized Anxiety Disorder-7, *UCLA Loneliness* The Japanese version of UCLA Loneliness Scale (Version 3), *VIA* Vancouver Index of Acculturation

### Structural validity

An item–total correlation analysis of the original 23 items showed no items with either excessively high (> 0.80) or low (< 0.30) item–total correlations, and therefore, no items were excluded [45].

MAP analysis supported a two-factor structure. An EFA specifying the two factors resulted in the exclusion of two items with excessively high uniqueness values (> 0.80). The final EFA yielded 21 items, with 11 and 10 items loading on the first and second factors, respectively. The first and second factors were labeled *Identity Discrepancy* and *Values Discrepancy*, respectively (Table 2).

**Table 2:** Pattern matrix and descriptive statistics for ADS-KJ among youth of Korean descent in Japan

Item	Factor1	Factor2	h2
Factor 1: Identity Discrepancy(McDonald's ω= .91)			
I perceive differences with my parents regarding which national identity they relate to.	.83	−.06	.64
I perceive differences with my parents regarding which country's history they are interested in.	.79	−.10	.56
I perceive differences with my parents regarding which traditional culture they prefer.	.77	−.08	.54
I perceive differences with my parents regarding which country's politics they are interested in.	.73	−.01	.52
I perceive differences with my parents regarding how much pride they feel in their nationality.	.70	.06	.53
I perceive differences with my parents regarding the ethnic background of their close friends.	.69	.04	.50
I perceive differences with my parents regarding which country's food they prefer.	.67	.11	.53
I perceive differences with my parents regarding which country they support in sports.	.64	.09	.48
I perceive differences with my parents regarding which language they use in daily life.	.61	−.05	.34
I perceive differences with my parents regarding the ethnic background of the person they would prefer me to marry.	.60	.09	.42
I perceive differences with my parents regarding whether I should use an ethnic name or a commonly used name.	.58	.05	.36
Factor 2: Value Discrepancy(McDonald's ω= .90)			
I perceive differences with my parents regarding the belief that "men must be active in society."	−.05	.84	.74
I perceive differences with my parents regarding the belief that "fathers should be the most respected in the family."	−.01	.81	.71
I perceive differences with my parents regarding the belief that "housework is for women."	−.01	.78	.59
I perceive differences with my parents regarding the belief that "children should attend high-ranking schools."	.00	.72	.49
I perceive differences with my parents regarding the belief that "parents should intervene in children's employment and education."	.08	.67	.52
I perceive differences with my parents regarding the belief that "children should work for prestigious companies."	−.02	.67	.44
I perceive differences with my parents regarding the belief that "children should be filial to their parents."	−.01	.60	.32
I perceive differences with my parents regarding the belief that "children should live with their parents even after marriage."	.03	.60	.34
I perceive differences with my parents regarding the belief that "parents should intervene in their children's romance or marriage."	.06	.59	.36
I perceive differences with my parents regarding the belief that "a mismatch between assigned and identified gender should not exist."	.05	.58	.33

Note.h2= communalities

The CFA results indicated an excellent model fit: CFI = 0.97, TLI = 0.97, SRMR = 0.05, and RMSEA = 0.05. Table 2.

### Internal consistency and Test–Retest reliability

For internal consistency, McDonald’s *ω* coefficients were *ω* = 0.91 for Identity Discrepancy and *ω* = 0.90 for Values Discrepancy (Table 2).

For test–retest reliability, *ICC(2*,*1)* values were: 0.93 for Identity Discrepancy (excellent), and 0.50 for Values Discrepancy (moderate).

### Construct validity

For convergent validity, the correlations between ADS-KJ subscale scores and scores on the PHQ-9, GAD-7, and UCLA Loneliness Scale were examined among Participants of Korean descent in Japan. Significant weak positive correlations were observed between the ADS-KJ subscale scores and PHQ-9, GAD-7, and UCLA Loneliness scores (Table 3).

**Table 3:** Intercorrelations among ADS-KJ factors and related variables in the sample of Korean descent in Japan

	1	2	3	4	5	6	7
1. Identity Discrepancy	−						
2. Values Discrepancy	0.48^**^	−					
3. VIA-Heritage	− 0.04	0.00	−				
4. VIA-Mainstream	− 0.13^*^	− 0.18**	0.19^**^	−			
5. UCLA Loneliness	0.26^**^	0.26**	− 0.25^**^	− 0.44^**^	−		
6. GAD-7	0.26^**^	0.30**	− 0.07	0.11	0.52^**^	−	
7. PHQ-9	0.30^**^	0.31**	− 0.11	^−^0.13^*^	0.52^**^	0.77^**^	−

Note. *p < .05. **p < .01

For discriminant validity, no significant correlations were observed between the ADS-KJ subscale scores and PHQ-9, GAD-7, or UCLA Loneliness scores among Japanese participants (Table 4).

**Table 4:** Intercorrelations among ADS-KJ factors and related variables in the Japanese sample

	1	2	3	4	5
1. Identity Discrepancy	−				
2. Values Discrepancy	.43**	−			
3. UCLA Loneliness	.13	.14	−		
4. GAD-7	.17	.13	.47**	−	
5. PHQ-9	.12	.08	.47**	.77**	−

Note. Identity Discrepancy: ADS-KJ Factor 1, Values Discrepancy: ADS-KJ Factor 2, *UCLA Loneliness* The Japanese version of UCLA Loneliness Scale (Version 3),  *GAD-7* The Japanese version of Generalized Anxiety Disorder-7, *PHQ-9* The Japanese version of Patient Health Questionnaire-9

*p < .05. **p < .01

### Criterion-related validity

Among participants of Korean descent in Japan, no significant correlations were observed between the ADS-KJ subscale scores and VIA–Heritage scores, while significant weak negative correlations were observed between the ADS-KJ subscale scores and VIA–Mainstream scores (Table 3).

## Discussion

This study developed and validated the ADS-KJ, a measure designed to assess youths’ subjective perceptions of parent–child acculturation discrepancies. The results supported a two-factor structure comprising Identity Discrepancy (11 items) and Values Discrepancy (10 items), yielding a final 21-item scale (Appendix 1 for the English version; Appendix 2 for the Japanese version).

### Structural validity

As hypothesized, the ADS-KJ demonstrated a two-factor structure consisting of 21 items assessing Identity Discrepancy and Values Discrepancy, with model fit indices indicating an excellent fit. The presence of discrepancies in cultural affiliation (Identity Discrepancy) and in values related to Confucian traditions (Values Discrepancy) is consistent with previous findings from studies of Asian American immigrant families in the United States [5]. Such acculturation discrepancies are likely to reflect cultural characteristics commonly observed among Asian immigrant families, including diverse affiliations that do not assume a singular national identity and strong cultural expectations regarding traditional family structures and filial piety [46].

In particular, items within the Identity Discrepancy factor related to historical and political perspectives concerning Korea and Japan appear to reflect acculturation discrepancies that are unique to youths of Korean descent in Japan, shaped by the historical context of Japanese colonial rule. For example, prior research has highlighted the major differences between Korean and Japanese history textbooks regarding topics, such as imperialization policies, comfort women, and forced labor during the colonial period [47]. These discrepancies in historical education may contribute to differences in historical understanding between youths and their parents, which, in turn, may foster perceptions of acculturation discrepancies.

### Internal consistency and test–retest reliability

For internal consistency, McDonald’s *ω* coefficients were above 0.90 for both subscales, indicating excellent internal consistency.

For test–retest reliability, *ICC(2*,*1)* was good for Identity Discrepancy and moderate for Values Discrepancy. The Values Discrepancy subscale includes several items related to major life transitions during emerging adulthood, such as school and career decisions, which may have been influenced by life changes that occurred between the two surveys. Given the participants’ mean age of 21.63 years (*SD* = 3.15), it is possible that some youth experienced events, such as conflicts with their parents regarding educational or career choices during the survey period, which may have led to greater variability in their responses on the Values Discrepancy subscale. In addition, the number of participants in the test–retest sample (*N* = 45) did not meet the recommended threshold of 100 or more participants [44]; thus, the results should be interpreted with caution. The response rate for the second survey may have been affected by the change in the survey format: the first survey was conducted in person, whereas the follow-up survey was conducted online.

### Construct validity

For convergent validity, significant positive correlations were observed between ADS-KJ subscale scores and PHQ-9, GAD-7, and UCLA Loneliness scores, supporting Hypothesis 2.

For discriminant validity, no significant correlations were found among the Japanese participants, supporting Hypothesis 3.

### Criterion-related validity

Hypothesis 4, which was based on the acculturation gap-distress model, was not supported in terms of criterion-related validity. No significant correlations were found between the ADS-KJ subscale scores and the VIA-Heritage scores, while significant weak negative correlations were found with the VIA-Mainstream scores. These results contradict the conventional premise that acculturation gaps arise when youths are more adapted to the mainstream culture and less adapted to the heritage culture than their parents. Instead, the perception of acculturation discrepancies appears to be associated with low adaptation to Japanese society and may occur independently of adaptation to the Korean heritage culture.

This seemingly paradoxical result can be systematically understood by considering the social and historical context surrounding youths of Korean descent in Japan. In this study, most participants were fourth-generation Oldcomers affiliated with ethnic community organizations. The philosophy of these organizations includes establishing legal status, stabilizing livelihoods, and promoting culture for youths of Korean descent in Japan who have long been socially oppressed [16]. Through social activities and historical education within these organizations, these youths are thought to be more acutely aware of the structural inequality and exclusion existing between themselves and Japanese society than their parents are. Consequently, they may be more prone to feeling psychological distance from Japanese society.

In contrast, their parents may have achieved higher levels of occupational and social adaptation through their extended experiences in Japanese society. As a result, gaps in adaptation to Japanese society may have emerged between youths and parents, which the youths perceived as acculturation discrepancies.

Thus, the acculturation discrepancy observed in this study does not represent the conventional objective gap where youths are more adapted to the mainstream society than their parents. Instead, it can be understood as a generational discrepancy in interpreting the meaning of their relationship with Japanese society. In other words, this gap may reflect differences in perceptions regarding the extent to which Japanese society is an accepting and fair space, and what appropriate distance should be maintained. Therefore, the ADS-KJ likely captures the perception of subjective distance formed within a social and historical context, which may explain why it showed a stronger association with psychological adaptation outcomes such as social isolation.

This point may also be understood through the framework of selective acculturation. Selective acculturation is the process by which immigrant groups maintain their ethnic culture while adopting specific cultural practices from the mainstream culture [48]. As shown in Table 1, the youths in this study are engaged in both heritage and mainstream cultures to some extent. However, the background of the acculturation gap may be that both parents and youths selectively adopt Japanese and Korean cultures across different domains, and these domains do not necessarily coincide. Future research needs to examine acculturation gaps across specific domains in greater detail.

### Clinical implications

Although some limitations regarding criterion-related validity remain, the ADS-KJ holds several important clinical implications for supporting Youth of Korean descent in Japan.

First, it enables the assessment of acculturation discrepancies as perceived by the youth themselves rather than relying on objective differences in acculturation levels. As such, it may be used in clinical settings as a screening tool to identify youths experiencing psychological distress at an early stage, thereby helping to prevent the development of more severe problems, such as depression and anxiety.

Second, the specific types of acculturation discrepancies identified through the ADS-KJ may convey the development of personalized intervention plans. For example, for youth who report high levels of discrepancy regarding historical and political views, practitioners could provide psychoeducation, that would help them to understand their parents’ perspectives in light of the historical context of colonial rule, thereby facilitating more effective support. In addition to psychoeducation on historical perspectives, family-based communication training or culturally informed counseling programs could also be implemented to address acculturation-related conflicts within Korean descent families in Japan.

Third, the ADS-KJ may contribute to the field of culturally informed assessment in Japan, where culturally sensitive assessment tools for immigrant populations remain scarce [49]. Given the increasing immigrant population in Japan, developing culturally informed approaches to provide psychological support is an urgent need. The ADS-KJ has the potential to enhance the quality of assessment and support for Youth of Korean descent in Japan, a group known to be at an elevated risk for psychological difficulties among immigrant populations in Japan.

### Study limitations and future directions

This study has several limitations that should be addressed in future research. First, unresolved issues related to test–retest reliability. The number of participants in the follow-up survey fell significantly below the threshold of 100 [44]. In addition, the potential influence of life changes on participants’ responses to the Values Discrepancy subscale may have affected the results. Future studies should control for the survey format, recruit a larger sample size, and consider using anchor-based methods to assess the stability of participants’ life circumstances [50].

Second, the limitations in criterion-related validity should be noted. While the ADS-KJ developed likely captures the perception of subjective distance formed within a social and historical context, we did not compare it with the conventional objective gaps in acculturation regarding their relationship with psychological adaptation. Future research should evaluate whether subjective or objective indicators better predict psychological adaptation, thereby enabling a more detailed examination of the psychological adaptation of youths of Korean descent in Japan.

Third, the conceptual clarity of the Identity Discrepancy subscale should be examined further. Some items may capture broader generational or sociopolitical differences that are not unique to Korean–Japanese acculturation; therefore, cultural specificity is not fully guaranteed. Future work should refine item content via cognitive interviewing, evaluate item functioning with IRT/DIF to identify and revise or replace non-specific items, and consider adding items that more explicitly situate identity-related discrepancies within the Korea–Japan historical and sociocultural context. Taken together, these steps will help ensure cultural specificity and strengthen the subscale’s construct validity.

Fourth, discriminant validity should be examined further. While the ADS-KJ demonstrated the ability to distinguish immigrant-related patterns from those of Japanese youths, its discriminant validity with respect to other immigrant groups has not been tested. It remains unclear whether the ADS-KJ captures acculturation discrepancies unique to Youth of Korean descent in Japan. Future research should compare Korean descent youth with other immigrant groups in Japan, to further evaluate discriminant validity.

Fifth, the generalizability of its findings is limited owing to the characteristics of the sample. Although sampling was conducted through ethnic organizations based on the anticipation that it would allow access to diverse participants [31], most participants were fourth-generation Oldcomers affiliated with these groups. This indicates that while the number of youths with diverse attributes, such as “doubles” and Newcomers, has been rapidly increasing in recent years, the proportion of Oldcomers within ethnic organizations remains high. Consequently, concerns remain regarding whether this scale is equally applicable to other migrant generations, such as Newcomers, or to youths not affiliated with ethnic organizations. Future studies should control for migrant generation and community affiliation and examine measurement invariance across subgroups.

## Conclusion

This study developed the ADS-KJ, which demonstrated generally good reliability and validity. However, limitations in criterion-related validity remain, and future research should assess both, youths’ adaptation to Japanese society, and their perceptions of their parents’ adaptation to it. The ADS-KJ enables a culturally informed assessment of youths’ subjective perceptions of acculturation discrepancies, and holds promise for further research and clinical applications. 

## Supplementary Information

Supplementary Material 1

Supplementary Material 2

## Data Availability

The datasets used and/or analyzed during the current study are available from the corresponding author on reasonable request.

## Abbreviations

ADS-KJ: Acculturation Discrepancy Scale for Youth of Korean Descent in Japan
PHQ-9: Patient Health Questionnaire-9
GAD-7: Generalized Anxiety Disorder-7
VIA: Vancouver Index of Acculturation
MAP: Minimum Average Partial
EFA: Exploratory Factor Analysis
CFA: Confirmatory Factor Analysis
CFI: Comparative Fit Index
TLI: Tucker–Lewis Index
SRMR: Standardized Root Mean Square Residual
RMSEA: Root Mean Square Error of Approximation
COSMIN: Consensus-based Standards for the Selection of Health Measurement Instruments
ICC: Intraclass Correlation Coefficient

## References

[1] Ferguson GM. The big difference a small Island can make: how Jamaican adolescents are advancing acculturation science. Child Dev Perspect. 2013;7(4):248–54. 10.1111/cdep.12051.

[2] Telzer EH, Yuen C, Gonzales N, et al. Filling gaps in the acculturation gap-distress model: heritage cultural maintenance and adjustment in Mexican–American families. J Youth Adolesc. 2016;45:1412–25. 10.1007/s10964-015-0408-8. 26759225 10.1007/s10964-015-0408-8

[3] Bámaca-Colbert MY, Henry CS, Perez-Brena N, Gayles JG, Martinez G. Cultural orientation gaps within a family systems perspective. J Fam Theory Rev. 2019;11:524–43. 10.1111/jftr.12353. 32405325 10.1111/jftr.12353 PMC7220130

[4] Szapocznik J, Kurtines WM. Family psychology and cultural diversity: opportunities for theory, research, and application. Am Psychol. 1993;48:400–7. 10.1037/0003-066X.48.4.400.

[5] Kim M, Park IJK. Testing the moderating effect of parent–adolescent communication on the acculturation gap–distress relation in Korean American families. J Youth Adolesc. 2011;40(12):1661–73. 10.1007/s10964-011-9648-4. 21404109 10.1007/s10964-011-9648-4 PMC3123726

[6] Rahman A, Sanchez M, Bursac Z, Cano MÁ. Acculturation gap conflicts and self-rated health among Hispanic emerging adults. Fam Syst Health. 2023;41(1):78–84. 10.1037/fsh0000726. 35951422 10.1037/fsh0000726 PMC11389867

[7] Sayers JL. Ambivalence as a potential mediator of associations between the parent-child acculturation gap and both depression and self-esteem in adolescent children of Mexican immigrant parents. Greensboro: University of North Carolina at Greensboro; 2016.

[8] Schwartz SJ, Unger JB, Baezconde-Garbanati L, Zamboanga BL, Cordova D, Lorenzo-Blanco EI, et al. Testing the parent–adolescent acculturation discrepancy hypothesis: A five-wave longitudinal study. J Res Adolesc. 2015;26(3):567–86. 10.1111/jora.12214. 27616871 10.1111/jora.12214 PMC5014429

[9] Shukla N, Kim SY, Telzer EH, et al. When and how do parent–child acculturation gaps matter? A systematic review and recommendations for research and practice. Clin Psychol Rev. 2025;102:102568. 10.1016/j.cpr.2025.102568. 10.1016/j.cpr.2025.102568 40073496

[10] Smokowski PR, Rose R, Bacallao ML. Acculturation and Latino family processes: how cultural involvement, biculturalism, and acculturation gaps influence family dynamics. Fam Relat. 2008;57(3):295–308. 10.1111/j.1741-3729.2008.00501.x.

[11] Unger JB, Ritt-Olson A, Wagner KD, Soto DW, Baezconde-Garbanati L. Parent–child acculturation patterns and substance use among Hispanic adolescents: A longitudinal analysis. J Prim Prev. 2009;30(3–4):293–313. 10.1007/s10935-009-0178-8. 19384604 10.1007/s10935-009-0178-8 PMC3739280

[12] Gilmour S, Hoshino H, Dhungel B. Suicide mortality in foreign residents of Japan. Int J Environ Res Public Health. 2019;16(17):3013. 10.3390/ijerph16173013. 31438491 10.3390/ijerph16173013 PMC6747213

[13] Kim T. Research on suicide prevention for Zainichi koreans: A compilation of questionnaire survey results. Bull Fac Sociol Toyo Univ. 2022;59(2):157–66.

[14] Kasuya K, Namiki M, Hayashi Y. Modern history of Korea. Tokyo: Yamakawa Shuppansha; 2016.

[15] Mizuno N, Moon K. Zainichi koreans: history and the present. Tokyo: Iwanami Shoten; 2015.

[16] Oh Y. History of education in Korean schools: struggles and creativity toward decolonization. Tokyo: Akashi Shoten; 2019.

[17] Kwak K. Hate speech against Zainichi Koreans and ideological interpellation: a reading through Judith butler’s theory of subjectivation. Jpn J Contemp Sociol Theory. 2014;8:39–54.

[18] Izawa T. Multiple identities and mental illness among Zainichi koreans: A life-history study of a man who is half Korean and a sexual minority. Bull Inst Hum Sci Toyo Univ. 2017;19:167–85.

[19] Ogose N. Language use among newcomer Zainichi Korean children: A survey conducted at an ethnic school in Tokyo. Jpn Stud. 2011;50:123–39.

[20] Song KC. The rise of the young generation of Zainichi Koreans and the new development of ethnic education. Kyoto J Sociol. 2001;9:237–53.

[21] Nukaga M, Shibano J, Miura A. Thinking about education from the perspective of migration: issues for children in the global era. Kyoto: Nakanishiya Publishing; 2019.

[22] Yoon SS. Interpersonal experiences of Zainichi Korean youth: A grounded theory analysis of narratives. Jpn J Educ Psychol. 2016;63(4):492–504.

[23] Kanazawa A. Exploring adolescent crisis and parent–child relationships among Zainichi Koreans (Koreans in Japan): A study of high school students. Psyche Cult. 2012;11(1):73–80.

[24] Morimoto H, Kishita N, Kondo H, Tanaka N, Abe Y, Muto T. Reliability and validity of the Japanese version of the experiential avoidance in caregiving questionnaire (EACQ). J Contextual Behav Sci. 2023;27:160–9. 10.1016/j.jcbs.2023.02.003.

[25] Kawakita J. The method of Idea generation: for the development of creativity. Tokyo: Chuokoron-shinsha; 1967.

[26] Scupin R. The KJ method: A technique for analyzing data derived from Japanese ethnology. Hum Organ. 1997;56(2):233–7. 10.17730/humo.56.2.x335923511444655.

[27] Ikeda H, Misawa R. Conceptualization and measurement of beliefs about failure. Jpn J Educ Psychol. 2012;60:367–79.

[28] Tatsumi Y, Haneshiri M, Nakamura N, Toume M, Tsuneto S, Kashiwagi T, et al. Extraction and new assessment scale development of family needs in ICU. Jpn Soc Intensive Care Med. 2005;12:111–8.

[29] Ryder AG, Alden LE, Paulhus DL. Is acculturation unidimensional or bidimensional? A head-to-head comparison in the prediction of personality, self-identity, and adjustment. J Pers Soc Psychol. 2000;79(1):49–65. 10.1037/0022-3514.79.1.49. 10909877 10.1037//0022-3514.79.1.49

[30] Keeney S, Hasson F, McKenna HP. The Delphi technique in nursing and health research. Chichester: Wiley-Blackwell 2011. 10.1002/9781444392029

[31] Ito M. Clinical psychology of adolescence and youth. Tokyo: Asakura Publishing; 2006.

[32] Kinoshita Y. What is the contemporary significance of ethnic organizations for Zainichi koreans? A case study of participants in the Korean youth association in Japan. J Soc Cult Stud Kumamoto Univ. 2023;21:39–57.

[33] Rumbaut RG. Severed or sustained attachments? Language, identity, and imagined communities in the post-immigrant generation. In: Levitt P, Waters MC, editors. The changing face of home: the transnational lives of the second generation. New York: Russell Sage Foundation; 2002. pp. 43–95.

[34] Muramatsu K, Miyaoka H, Kamijima K, Muramatsu Y, Tanaka Y, Hosaka M, et al. Performance of the Japanese version of the patient health Questionnaire-9 (J-PHQ-9) for depression in primary care. Gen Hosp Psychiatry. 2018;52:64–9. 10.1016/j.genhosppsych.2018.03.007. 29698880 10.1016/j.genhosppsych.2018.03.007

[35] Muramatsu K, Miyaoka H, Ueshima K, Muramatsu Y, Fuse K, Yoshimine F, et al. Validation and utility of the Japanese version of the generalized anxiety disorder 7-item scale (GAD-7). Jpn J Psychosom Med. 2010;50(6):592.

[36] Masuda Y, Tadaka E, Dai A. Development and validation of the Japanese version of the UCLA loneliness scale (Version 3) among older adults. Jpn J Acad Community Health Nurs. 2012;15(1):25–32.

[37] Toyoshima A, Sato M. Development of a short form of the Japanese version of the UCLA loneliness scale (Version 3): toward use across generations. Jpn J Clin Geropsychol. 2021;2:19–25.

[38] Hwang WC, Wood JJ. Acculturative family distancing: links with self-reported symptomatology among Asian Americans and Latinos. Child Psychiatry Hum Dev. 2009;40(1):123–38. 10.1007/s10578-008-0115-8. 18663569 10.1007/s10578-008-0115-8

[39] Kim SY, Chen Q, Wang Y, Shen Y, Orozco-Lapray D. Longitudinal linkages among parent–child acculturation discrepancy, parenting, parent–child sense of alienation, and adolescent adjustment in Chinese immigrant families. Dev Psychol. 2013;49(5):900–12. 10.1037/a0029169. 22799587 10.1037/a0029169 PMC3514557

[40] Birman D. Acculturation gap and family adjustment: findings with Soviet Jewish refugees in the united States and implications for measurement. J Cross Cult Psychol. 2006;37(5):568–89. 10.1177/0022022106290479.

[41] Hu LT, Bentler PM. Cutoff criteria for fit indexes in covariance structure analysis: conventional criteria versus new alternatives. Struct Equ Model. 1999;6(1):1–55. 10.1080/10705519909540118.

[42] Dunn TJ, Baguley T, Brunsden V. From alpha to omega: A practical solution to the pervasive problem of internal consistency Estimation. Br J Psychol. 2014;105(3):399–412. 10.1111/bjop.12046. 24844115 10.1111/bjop.12046

[43] Koo TK, Li MY. A guideline of selecting and reporting intraclass correlation coefficients for reliability research. J Chiropr Med. 2016;15(2):155–63. 10.1016/j.jcm.2016.02.012. 27330520 10.1016/j.jcm.2016.02.012 PMC4913118

[44] Guilford JP. Fundamental statistics in psychology and education. 3rd ed. New York: McGraw-Hill; 1956.

[45] Prinsen CA, Mokkink LB, Bouter LM, Alonso J, Patrick DL, de Vet HC, et al. COSMIN guideline for systematic reviews of patient-reported outcome measures. Qual Life Res. 2018;27:1147–57. 10.1007/s11136-018-1798-3. 29435801 10.1007/s11136-018-1798-3 PMC5891568

[46] Norman GR, Streiner DL. Health measurement scales: A practical guide to their development and use. 4th ed. Oxford: Oxford University Press; 2008.

[47] Chung RHG, Kim BSK, Abreu JM. Asian American multidimensional acculturation scale: Development, factor analysis, reliability, and validity. Cult Divers Ethn Minor Psychol. 2004;10(1):66–80. 10.1037/1099-9809.10.1.66. 10.1037/1099-9809.10.1.66 14992631

[48] Kano S, Tsuchiya T. The subject of the history textbook problem between Japan and Korea, and a view. Bull Aichi Univ Educ. 2002;5:25–32.

[49] Eitle TM, Wahl AM, Aranda E. Immigrant generation, selective acculturation, and alcohol use among Latina/o adolescents. Soc Sci Res. 2009;38(3):732–42. 10.1016/j.ssresearch.2009.01.006. 19856707 10.1016/j.ssresearch.2009.01.006 PMC2699685

[50] Yamaguchi T. Factors affecting the access and use of medical and health information by foreign residents in japan: A review of the relevant literature. Jpn J Nurs Health Sci. 2023;70(1):29–40. 10.20705/jjnhs.21.0_29.

